# Identifying child temperament risk factors from 2 to 8 years of age: validation of a brief temperament screening tool in the US, Europe, and China

**DOI:** 10.1007/s00787-019-01379-5

**Published:** 2019-08-14

**Authors:** Marcel Zentner

**Affiliations:** Department of Psychology, University of Innsbruck, Innrain 52, 6020 Innsbruck, Austria

**Keywords:** Child temperament, Measurement invariance, Behavior problems, Assessment, Screening

## Abstract

Despite ample evidence linking particular child temperament characteristics to behavior disorders later in life, there is currently a lack of temperament measures that can be used early, easily, and widely for screening purposes. To redress this gap, the current research aimed at developing a very brief scale of child temperament characteristics that have been found to predict behavior problems over the long term, are represented across models of temperament, and have the potential to exhibit measurement invariance over different countries and childhood periods. The new scale was derived from the Integrative Child Temperament Inventory, a 30-item measure to assess five well-established temperament dimensions, and examined in three studies with samples of children aged between 2 and 8 years across five countries: The United States, the United Kingdom, China, Germany, and Spain (*N* = 13,425; boys 55.96%). The studies included tests of measurements invariance, of convergent validity with established measures of temperament, and of criterion validity with measures of behavior problems. The scale exhibited full metric invariance and partial scalar invariance across age groups (toddlerhood, preschool, school age) and countries. Test–retest reliability, interrater reliability across teachers, and convergent and criterion validity were adequate. Preliminary data on the measure’s clinical utility suggest a favorable balance between brevity and screening accuracy. Altogether, this study suggests that early childhood temperament characteristics placing children at risk for developing behavior problems much later in life can be quickly, effectively, and commensurably assessed across different countries and age groups.

**Electronic supplementary material:**

The online version of this article (10.1007/s00787-019-01379-5) contains supplementary material, which is available to authorized users.

## Introduction

Temperament plays a significant role in shaping various outcomes, including parent–child interactions, attachment, scholastic achievement, adult personality, and psychopathology (for an overview, see [1, 2]. For example, children high on behavioral inhibition have up to seven times the risk of developing social anxiety disorder (SAD) as that of controls, making behavioral inhibition “a principal predictor of SAD” ([3], p. 1072). Poor self-control in preschool, in turn, has been found to predict adult antisocial disorders just as strongly as “low intelligence and low social class origins, which are known to be extremely difficult to improve through intervention” ([4], p. 2697). Despite its clinical significance, temperament plays a marginal role in child mental health settings [5].

One likely reason for this neglect lies in a disorientating array of child temperament measures and models that carry different names but often comprise constructs with considerable overlap. These have included a behavioral-stylistic [6], an emotion-related [7], a regulatory [8], a criterial [9], and two different psychobiological approaches [10, 11]. Since each of these approaches proposes its own set of measures, there are currently over 30 child temperament measures, largely questionnaires [12], and a few observational measures [13]. The items and dimensions included in current temperament measures not only vary across temperament models, they also vary across age periods within one and the same temperament model. Three to four age-specific versions of each instrument often exist, usually for the infancy, preschool, and school periods, sometimes supplemented by an instrument for toddlerhood and/or late childhood [12].

The existence of age-specific instruments is sensible given young children’s rapid rate of development and maturation. However, issues of comparability and commensurability arise when findings obtained via different instruments are compared across age groups. Although a few instruments such as the Emotionality, Activity, and Sociability (EAS) Temperament Survey for children [9] can be applied across a wider age range, there is little evidence on how, if at all, the measures are age invariant. A related question in the current child temperament literature is the comparability of temperament measures across cultures. The vast majority of temperament measures were developed in English, and widely used instruments were thereafter translated into other languages. To date, there are no studies that have examined measurement invariance of core child temperament factors in several countries and age groups contemporaneously, making previous findings vulnerable to the criticism that “cross-group comparisons on the factors have no meaning or interpretation” ([14], p. 547).

A more practical barrier to using child temperament measures widely is that even the shortest temperament questionnaires are comparatively long. For example, the “very short” form of the Children’s Behavior Questionnaire (CBQ) includes 36 items [15]. The 20-item EAS has no subscale related to effortful control and may still be too long for use in contexts that require assessments of numerous constructs along with measures of temperament, such as in large-scale studies where temperament is primarily measured as a control variable, or in primary pediatric care settings that screen for children’s behavioral or emotional risk.

To counteract these limitations, the current research aimed at developing a veritably brief measure of well-studied child temperament characteristics that are represented across models of temperament and that have been found to predict behavior problems over the long term. Whereas several preschool temperament characteristics have been linked to clinically significant outcomes in the short- and mid-term, few characteristics have been found to consistently predict adult outcomes in prospective longitudinal studies. Most of the evidence for persistent, long-lasting effects of infant-to-preschool temperament crystallizes around three components [16]. The first temperament component relates to irritability, frustration and anger proneness. The second one includes impairments in attentional and impulse control, sometimes also referred to as “undercontrol”, is positively related to novelty seeking, and negatively to persistence and effortful control. Both components are established predictors of externalizing problem behavior. The third component is behavioral i nhibition, which is related to harm avoidance, and is a well-known risk factor for the development of internalizing problem behavior [16]. Selected prospective longitudinal studies documenting the long-term predictive power of these temperamental components are summarized in Tables 1 and 2. Relationships of the three components to widely used temperament scales are shown in Supplementary Materials 1.

**Table 1:** Infant-to-preschool temperament predictors of adolescent and adult personality and psychopathology: undercontrol/inattention

Longitudinal study	Early childhood temperament	Adolescent/adult outcomes	Predictive range	Key references
Dunedin Health and Development Study	Undercontrol/impulsivity	Elevated suicide risk Criminal offending Substance dependence	3–18 years 3–26 years 3–32 years	Caspi et al. [17] Caspi et al. [18] Moffitt et al. [4]
Mauritius Child Health Project	Fearlessness, disinhibition	Psychopathy	3–28 years	Glenn et al. [19]
Block and Block Longitudinal Project	Ego-undercontrol	Ego-undercontrol Narcissism	3–23 years 3–23 years	Block and Block [20] Carlson and Gjerde [21]
Colorado Longitudinal Twin Study	Impulse control	Executive functions	18–36 months to 16–17 years	Friedman et al. [22]
Mannheim Longitudinal Study	Attentional deficits	Novelty seeking	3 months to 16 years	Laucht et al. [23]
Fullerton Longitudinal Study	Temperamental difficulty	Externalizing and internalizing behaviors	18 months to 17 years	Guerin et al. [24]

Tables 1 and 2 adapted from Ref. [25]

**Table 2:** Infant-to-preschool temperamental predictors of adolescent and adult personality and psychopathology: behavioral inhibition

Longitudinal study	Early childhood temperament	Adolescent/adult outcomes	Predictive range	Key references
Harvard Longitudinal Study	High reactivity	Trait anxiety Amygdala hyperresponsiveness	4 months to 15 years 4 months to 21 years	Kagan et al. [26] Schwartz et al. [27]
Dunedin Health and Development Study	Inhibition	Depression Harm avoidance	3–18 years 3–26 years	Caspi et al. [17] Caspi et al. [18]
LOGIC Study	Inhibition	Internalizing problems	4–23 years	Asendorpf et al. [28]
Uppsala Longitudinal Study	Shyness	Social anxiety Depressive symptoms	20 months to 21 years 20 months to 21 years	Bohlin and Hagekull [29]

The etiological sequence associated with these temperamental qualities appears to start before the third birthday and to be particularly long lived, making these qualities prime candidates for inclusion in a screening tool for early temperament risk factors. This does not mean that other temperamental factors, such as lack of positive emotion, or activity level, are necessarily of lesser clinical relevance. As more recent birth cohort studies that include measures of temperament approach the 20-year mark, the characteristics listed in Tables 1 and 2 may have to be revisited.

A second aim that guided the selection of traits to be included in the new measure was their potential to exhibit measurement invariance between toddlerhood and school age. The basis for the development of the measure was provided by the Integrative Child Temperament Inventory (ICTI), a 30-item measure to assess five well-established temperament dimensions [30]. One advantage of the ICTI is that it includes scales that assess the three previously mentioned clinically relevant temperamental qualities; another is that it spans a relatively wide age range, thereby lending itself to examinations of measurement invariance between toddlerhood and school age. The following sections briefly review the research literature regarding the three components as they relate to core definitional features of temperament (e.g., forms of expression, biological correlates, and stability across time), as well as to their clinical significance.

*Irritability, frustration, and anger* A cluster of three interrelated dimensions (irritability, frustration, and anger proneness) defines one of the most clinically significant components of child temperament [16, 31]. Broadly speaking, irritability refers to some infants being more easily upset by minor discomforts than others. Irritability is one of the key elements of the “difficult temperament” construct proposed by Thomas and Chess [6] and measured by the Infant Characteristics Questionnaire [32], where it is defined by frequent and intense negative affect and the degree of difficulty that the infant presents to caregivers. A slightly later emerging quality related to irritability and “difficultness” is frustration. It can be defined as a negative, predominately angry, affect in reaction to an externally imposed interruption of ongoing tasks or blocking of behaviors related to approach and goal attainment [31].

Irritability and frustration proneness may be related to dysfunctions in neural circuits involving the striatum, anterior cingulate cortex, amygdala, and parietal lobes, with panic and defensive aggression representing extreme examples of neurobehavioral dysregulation [33]. Infants’ level of anger was found to predict parent-reported externalizing problems when the children were 8 years old, even after controlling for initial levels of externalizing problems [34].

*Attention/persistence* Attentional focusing and persistence are key components of effortful control—an increasingly significant temperament concept relating to “regulatory” aspects of temperament [11, 35]. Effortful control has been defined as the ability to inhibit a dominant response and/or activate a subdominant response, to plan, and to detect errors [11]. Like anger/frustration, low effortful control has been found to predict various types of externalizing problems, including attention deficit hyperactivity disorder, substance dependence, conduct and antisocial personality disorders (e.g., [36–39]. The two dimensions often compound one another in putting children at risk for externalizing behavior problems. Thus, toddler inattention and impaired emotion regulation, as measured in response to a frustration task, were found to be powerful predictors of a chronic externalizing profile [40], and they also coalesce in the clinically significant construct of undercontrol (see Table 1).

Effortful control can be differentiated into two major subcomponents: (a) “attentional control,” which is the capacity to maintain attention on tasks and to shift attention when desired, and (b) “inhibitory control,” which is the capacity to plan and to suppress inappropriate action. Posner et al. [41] identified the frontoparietal network as supporting the former component and the dorsolateral prefrontal cortex, the anterior cingulate cortex, and cingulo-opercular network as supporting the latter. Attentional focusing and inhibitory control have both been found to predict later outcomes. For example, attention problems of 3-month old infants have been shown to predict novelty seeking in adolescence [23]. Preschool delay of gratification, which is related to inhibitory control, has been found to predict cognitive and self-regulatory competencies in adolescence (e.g., [20, 42]. It is important to note that attentional focusing/persistence develops earlier than does inhibitory control and that it has also been found to be the more stable dimension of the two across childhood [43]. For this reason, attentional focusing and attentional persistence are more promising components to include in a measure designed to be measurement-invariant over various childhood periods than is inhibitory control.

*Behavioral inhibition* Behavioral inhibition to the unfamiliar and its related characteristics (e.g., shyness, approach/withdrawal, harm avoidance) are included in virtually all child temperament models and questionnaires [44]. Although behavioral inhibition has a relatively broad meaning that may include avoidance of physical risks and inhibition in evaluative situations [24], its most frequent expression is social fearfulness. It is important to distinguish behavioral inhibition from inhibitory control. The former is reactive and results from relatively automatic fear or distress responses in new situations. In contrast, the latter involves the regulatory use of executive attention and expresses itself in behaviors such as resisting temptation or delaying gratification [45]. Behavioral inhibition and its infancy precursors have been identified as risk factors for the development of anxiety and depressive disorders (e.g., [3, 46, 47] see also Table 2).

Hyperresponsiveness of the amygdala appears to promote behavioral inhibition [27], but connectivity to other brain areas such as the anterior cingulate can moderate this link [48]. In early infancy, behavioral inhibition tends to be expressed by the degree of tenseness, motor activity, and crying shown in response to the unexpected appearance of unfamiliar visual, auditory, or olfactory stimuli [49], and these patterns of reactivity have been shown to be moderately stable between infancy and adolescence [26, 46].

## The current studies

The preceding review of temperament characteristics and correlates provides the background for the development of the measure to be described next. Drawing on parent and caretaker ratings of toddlers, preschoolers, and early school-age children from the United States, the United Kingdom, Germany, Spain, and China, Study 1 describes the development of this measure from its parent instrument, the ICTI, its internal structure, measurement invariance, and selected validity indicators, including the EAS and the CBQ for examining convergent validity, and a four-item measure of perceived child difficulty for testing criterion validity. Measurement invariance was examined with multigroup confirmatory factor analysis. Studies 2a and 2b examined forms of reliability other than internal consistency: retest reliability, interrater reliability in parents (Study 2a), and interrater reliability among preschool teachers (Study 2b). Study 3 explored the scale’s clinical usefulness in detecting children exhibiting externalizing and internalizing problem behaviors based on their temperament characteristics.

## Study 1: factor structure, measurement invariance, and validity

### Methods

*Sample and procedures* Participants were parents and childcare professionals who completed an online questionnaire on children’s temperament by visiting a website specifically devised for the purpose of this research. The site, which existed in a German language, an English language, a Spanish language, and a Mandarin Chinese language version, offered general information about child temperament and invited the visitors to provide a temperament rating of their child if she/he fell within the suitable age range (2–8 years). The study was approved by the departmental ethics committee and participants provided informed consent before taking the survey. As part of the survey, participants provided information about the age, gender, and nationality of the child, as well as about their own age, gender, nationality, and educational attainment. To help raise awareness of the survey in as diverse a population as possible, Google AdWords advertisements were placed in each nation. Standard procedures for quality control of Internet data were followed (see [50]. Thus, multiple entries from the same participants were removed and respondents who entered the same number more than 12 times in succession were also removed. Table 3 provides descriptive information of the samples in the first two rows of each sample section.

**Table 3:** Sample sizes and mean ages for girls and boys, means and standard deviations of the ICTS scales, effect sizes for gender differences, Cronbach’s *α*, and McDonald’s *ω*

Sample	Overall	Boys	Girls	*d*	*α*	$\omega$
US sample, *N*	3491	1923	1568			
Mean age (SD)^a^	55.24 (21.63)	55.62 (21.42)	54.77 (21.88)			
Frustration	12.40 (3.38)	12.46 (3.38)	12.32 (3.38)	0.04	0.74	0.74
Inhibition	9.46 (4.16)	9.28 (4.09)	9.68 (4.23)	− 0.10**	0.81	0.83
Attention	11.22 (3.16)	11.10 (3.25)	11.37 (3.05)	− 0.09**	0.74	0.74
UK sample, *N*	730	411	319			
Mean age (SD)	59.31 (23.39)	59.60 (23.21)	58.95 (23.64)			
Frustration	12.00 (3.37)	11.91 (3.43)	12.10 (3.29)	− 0.06	0.64	0.65
Inhibition	9.55 (3.88)	9.48 (6.16)	9.65 (6.56)	− 0.04	0.72	0.75
Attention	10.77 (3.36)	10.63 (3.40)	10.96 (3.29)	− 0.10	0.73	0.74
German sample, *N*	4409	2446	1963			
Mean age (SD)	60.48 (23.12)	61.74 (23.14)	58.95 (23.64)			
Frustration	12.11 (3.40)	12.45 (3.30)	11.67 (3.47)	0.23**	0.69	0.70
Inhibition	9.53 (4.08)	9.32 (4.02)	9.79 (4.14)	− 0.11**	0.76	0.79
Attention	10.35 (3.44)	10.19 (3.38)	10.56 (3.50)	− 0.11**	0.72	0.73
Spanish sample, *N*	1448	839	609			
Mean age (SD)	48.56 (19.93)	48.87 (20.18)	48.14 (19.79)			
Frustration	10.42 (3.95)	10.58 (3.98)	10.20 (3.90)	0.09	0.78	0.79
Inhibition	9.31 (3.36)	9.12 (3.96)	9.59 (3.93)	− 0.12*	0.73	0.76
Attention	11.09 (3.59)	10.83 (3.62)	11.45 (3.52)	− 0.17**	0.74	0.74
Chinese sample, *N*	2669	1517	1152			
Mean age (SD)	56.45 (23.84)	56.62 (23.79)	56.23 (23.92)			
Frustration	10.00 (3.71)	10.27 (3.69)	9.63 (3.70)	0.17**	0.68	0.72
Inhibition	8.89 (3.70)	8.64 (3.65)	9.22 (3.74)	− 0.16**	0.71	0.73
Attention	11.52 (3.42)	11.26 (3.37)	11.87 (3.47)	− 0.18**	0.68	0.68

*ICTS* Integrative Child Temperament Screener, *d *effect sizes for gender differences in Cohen’s *d* units (significance levels refer to *t *tests for the difference between girls and boys, *p < 0.05, **p < 0.01); *α* = Cronbach’s *α*; *ω* = McDonald’s omega

^a^Age in months

### Measures

*Integrative Child Temperament Questionnaire (ICTI)* The ICTI is a 30-item measure to assess five well-researched temperament dimensions: anger/frustration, behavioral inhibition, attention/persistence, activity level, and sensory sensitivity in children between 2 and 8 years of age [30]. This age range was chosen because (a) it covers a key period for the assessment of early temperament risk factors, and (b) it spans a relatively wide range, extending from toddlerhood to early school age, all while (c) allowing for using the same items for behaviors at the early and the late end of the range. The instrument was originally developed and validated in a sample of German participants (see [51], followed by an adaptation to UK and US populations [30]. The methods used in the construction and validation of the original instrument are covered in Zentner and Wang [30]. Broadly, items and scales were generated according to converging views on important domains of child temperament [1, 2, 16, 36, 52], and following established item-analytic procedures, such as described in De Vellis [53].

To derive the current screening instrument, the psychometric analyses focused on the three clinically most significant scales of the ICTI (i.e., anger/frustration, behavioral inhibition, and attentional persistence; see the “Introduction”). From their psychometric merits, their likelihood of exhibiting measurement invariance over the instrument’s age range, and their suitability for screening in school, home, and pediatric care contexts, three items per dimension were chosen for an in-depth analysis and potential inclusion in the new measure. The nine items are reproduced in the “Appendix”. In reference to the ICTI, the scale is henceforth referred to as the Integrative Child Temperament Screener (ICTS). For the sake of brevity, the ICTS dimensions will sometimes simply be referred to as frustration (for anger/frustration), inhibition (for behavioral inhibition) and attention (for attentional persistence). Two bilingual native speakers translated the items of the English version into Spanish and Mandarin, and two others provided the back-translation. Two additional bilingual speakers resolved any discrepancies between the original version and the back-translations.

*EAS temperament survey for children (parental rating form)* The EAS questionnaire is a widely used and validated measure of temperament for children aged 1–12 years [9]. The scales emotionality and shyness were used to examine convergent validity with the ICTS frustration and inhibition. Since the EAS has no component related to attentional control and persistence, the scale “attention span/persistence” from the Colorado Child Temperament Inventory [54] was used to examine convergent validity with ICTS attentional persistence. These scales were included in the US, the UK, and the German samples.

*Children’s Behavior Questionnaire-Short Form (CBQ-SF)* The CBQ is an extensively validated parent report measure of temperament for children 3–7 years old [55]. The scales anger, shyness, and attentional focusing of the short form were used to examine convergent validity with the ICTS frustration, inhibition, and attention in the US, UK, and Chinese samples.

*Perceived child difficulty* For the purposes of examining criterion validity, parents answered four questions about difficulties with their child: (a) frequency of being irritated by the child, (b) frequency of being disappointed by the child, (c) perceived difficulty of child rearing, and (d) global difficulty rating of the child. The answer format consisted of six-point scales ranging from never or almost never to always or almost always for items a–c, and from very easy to very difficult for item d. The composite computed across these four items was internally consistent (United States: *α *= 0.88; United Kingdom: *α* = 0.88; Germany: *α* = 0.87; Spain: *α* = 0.84; China: *α* = 0.71).

### Results

*Descriptive statistics* A comparison of the samples’ educational attainment with representative data from census or census-type statistics indicated that participants were somewhat more educated than the general population (see Supplementary Materials 2). Means and standard deviations of the scales for boys and girls, as well as internal consistencies for all five samples, are reproduced in Table 3. In addition to Cronbach’s *α*, McDonald’s *ω* was used to estimate internal consistency because of its more realistic underlying assumptions [56]. Tests of gender differences are shown in the third column from the right. As in previous studies, attentional persistence was consistently higher in girls compared to boys [57]. The associations between age (in months) and scores on the three temperament dimensions were small overall (all *r*s ≤ 0.20), and none of the associations were consistent across the five samples.

Correlations between the full ICTI-scales and the short ICTS scales were all *r* ≥ 0.90. Of special interest is the ICTI attention/persistence scale, because the retained three items related to attentional persistence, whereas the omitted three items were behavioral persistence items. Behavioral persistence is conceptually and empirically related to inhibitory control—a key facet of effortful control next to attentional persistence and focusing [43]. The correlation between the three averaged behavioral persistence items and the ICTS attentional persistence scale computed across the full sample was *r* = 0.67 (*p* < 0.001), suggesting a close relationship between ICTS attentional persistence and effortful control.

*Internal structure and measurement invariance* The measurement model to be examined consisted of three latent factors (frustration, inhibition, and attentional persistence), each represented by three items as the observed variables. Due to previous findings suggesting a strong negative relationship between frustration and effortful control, the latent factor frustration and attentional persistence were allowed to correlate with one another. Measurement invariance was examined across (a) the samples from the five countries and (b) three age groups that were formed so as to include about an equal number of toddlers (2.0–3.5 years, *N* = 4376), preschoolers (3.5–5.5 years, *N* = 4118), and school-age children (5.5–8.0 years, *N* = 4253). Tests of invariance involved the progressive comparison of nested models, increasingly constrained from configural to metric, and then from metric to scalar invariance. The model was examined with R v3.5.0, using maximum likelihood estimation with robust (Huber–White) standard errors. As the scales showed some skew, the Yuan–Bentler scaling correction was applied. The proposed three-indicator, three-factor model fit the data well overall, as can be seen from Table 4.

**Table 4:** Model fit indices for configural measurement invariance of the ICTS across countries (upper part) and age groups (lower part)

Sample(s)	CFI	SRMR	RMSEA [90% CI]	*χ*^2^ (*df*)*
Countries				
US (single group)	0.976	0.051	0.049 [0.043, 0.055]	225.34 (26)
UK (single group)	0.962	0.051	0.053 [0.039, 0.670]	76.63 (26)
Germany (single group)	0.972	0.033	0.049 [0.044, 0.054]	276.43 (26)
China (single group)	0.941	0.055	0.066 [0.059, 0.072]	295.43 (26)
Spain (single group)	0.977	0.040	0.048 [0.038, 0.058]	104.41 (26)
All nations (multiple groups, no equality constraints)	0.968	0.040	0.052 [0.049, 0.055]	936.62 (108)
Age groups				
Toddlers	0.982	0.034	0.039 [0.034, 0.044]	181.38 (26)
Preschoolers	0.983	0.036	0.039 [0.034, 0.045]	177.01 (26)
School-age children	0.980	0.042	0.042 [0.037, 0.048]	207.31 (26)
All ages (multiple groups, no equality constraints)	0.982	0.034	0.040 [0.037, 0.043]	565.50 (78)

*ICTS* Integrative Child Temperament Screener, *CFI *comparative fit index, *SRMR* standardized root mean square residual, *RMSEA *root mean square error of approximation, *CI *confidence interval

*All *p* values associated with the *χ*^2^ test = *p* < 0.01

Following Cheung and Rensvold’s recommendations [58], the presence of invariance at each level of model constraint was evaluated using changes (Δ) in fit indices, rather than changes in *χ*^2^, between a more restricted model and the preceding one. The general recommendation is to use Δ root mean square error of approximation (RMSEA) ≤ 0.015 and Δ comparative fit index (CFI) ≤ 0.01 as criteria for the tenability of invariance [59]. These criteria were typically validated for two-group investigations, however. Based on the work by Rutkowski and Svetina [60], the OECD has adopted ΔRMSEA ≤ 0.030 and ΔCFI ≤ 0.02 as more realistic criteria for evaluating the presence of metric invariance, particularly when comparisons are carried out across a larger number of groups [61].

As can be seen from Table 5, metric invariance was attained for the different age groups. To ensure that the results would not depend on the particular age break points used, invariance analyses for age were run on a number of alternative age groups (e.g., 2.0–3.0 years; 3.1–5.5 years; 5.6–8.0 years) that yielded similar findings to those reported in Table 5 (these analyses are available upon request). For countries, the metric invariance model held up against any of the above criteria for ΔRMSEA; ΔCFI was within the bounds of the criterion suggested by the OECD.

**Table 5:** Model fit indices for metric and scalar measurement invariance of the ICTS across countries (upper part) and age groups (lower part)

Model	CFI	SRMR	RMSEA [90% CI]	*χ*^2^ (*df*)*	Δ CFI	Δ RMSEA
Countries						
Configural invariance	0.968	0.040	0.052 [0.049, 0.055]	936.62 (108)	–	–
Metric invariance	0.949	0.061	0.059 [0.056, 0.062]	1561.90 (166)	0.019	− 0.006
Scalar invariance	0.900	0.071	0.078 [0.075, 0.080]	2956.41 (190)	0.048	− 0.018
Partial scalar invariance	0.942	0.063	0.061 [0.058, 0.063]	1794.27 (182)	0.007	− 0.002
Age groups						
Configural invariance	0.982	0.034	0.040 [0.037, 0.043]	565.50 (78)	–	–
Metric invariance	0.978	0.041	0.039 [0.037, 0.042]	685.75 (96)	0.004	0.001
Scalar invariance	0.927	0.053	0.068 [0.066, 0.071]	2111.40 (108)	0.051	− 0.029
Partial scalar invariance	0.970	0.043	0.045 [0.042, 0.047]	917.57 (102)	0.008	− 0.006

*ICTS *Integrative Child Temperament Screener, *CFI* comparative fit index, *SRMR *standardized root mean square residual, *RMSEA *root mean square error of approximation, *CI *confidence interval

*All *p *values associated with the *χ*^2^ test = *p* < 0.01

Full scalar invariance was found for neither countries nor age groups. Thus, modification indices were inspected to identify the thresholds that needed to be freed in view of examining partial invariance. With regard to age, partial scalar invariance was attained by freeing the equality of intercept constraint for the first frustration item, the first attentional persistence item, and the second inhibition item leaving two invariant item intercepts per factor. With regard to countries, partial scalar invariance was attained by freeing the first frustration and the first attentional persistence items. The internal consistency reliabilities of the invariant two-item subscales computed across all nations were: *ω* = 0.71 for frustration, *ω* = 0.76 for behavioral inhibition, and *ω* = 0.63 for attentional persistence.

**Fig. 1:**
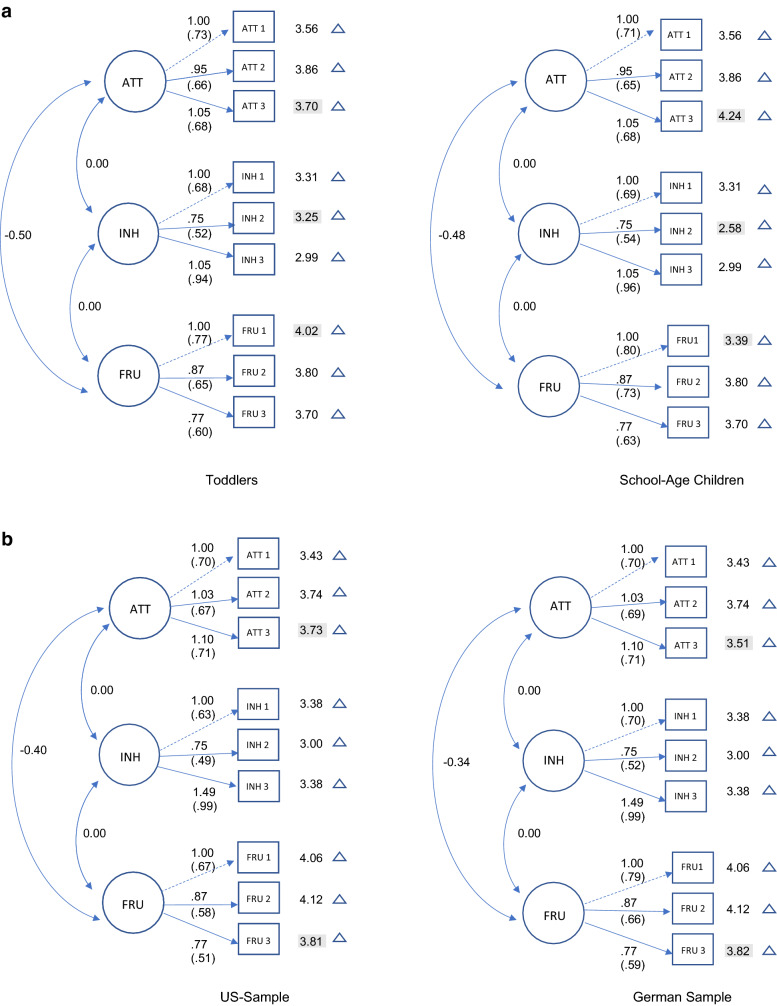
**a** Final measurement model for scalar invariance across two age groups: toddlers and school-age children.** b** final measurement model for scalar invariance across two countries, US and Germany. Values represent covariances, factor loadings, and item intercepts. Values are unstandardized. Standardized factor loadings are given in parentheses. Highlighted intercepts were freed to attain partial scalar invariance. *ATT* attentional persistence, *INH* behavioral inhibition, *FRU* anger/frustration (see Supplementary Table 3 for complete parameter estimates)

Figure 1 shows the results of the final measurement model, with scalar invariance parameter estimates across two age groups (toddlerhood and early school age, see Fig. 1a) and two countries (Germany and the US, see Fig. 1b). Detailed parameter estimates for all nations and age groups are provided in Supplementary Materials 3.

*Relation to other measures* The correlations with the temperament scales chosen for investigating convergent validity are reported in Table 6, with boldfaced values highlighting the expected validity correlations. Since differences in the correlations were small between the US and the UK samples, the two samples are combined in Table 6 for economy of presentation. To keep the rating sessions within reasonable limits, not all validation instruments were given to all participants. In the German sample, convergent validity was examined with the EAS, whereas in the Chinese sample it was examined with the CBQ-SF only. The respective *N*s are reported in the note to Table 6. Criterion validity was examined against perceptions of child difficulty (see “Methods”), which have consistently been found to relate to negative emotionality, irritability, anger, and frustration, as well as to lack of effortful control [39]. Consistent with these findings, the highest correlations with the child difficulty scale were found for frustration, *r* = 0.41 (China) to *r* = 0.69 (Spain), followed by attentional persistence, *r* = − 0.31 (China) to *r* = − 0.40 (Germany), and inhibition (all *r*s ≤ 0.15; see Supplementary Materials 4 for details). Taken together, these findings attest to the ICTS’s convergent- and criterion-related validity.

**Table 6:** Convergent validity: correlations of ICTS dimensions with related dimensions of the CBQ-SF and the EAS in four samples (US, UK, Germany, China)

	Frustration	Inhibition	Attention
US/UK sample^a^			
CBQ anger	0.60*	0.06	− 0.29*
CBQ shyness	0.12*	0.84*	− 0.00
CBQ attentional focusing	− 0.30*	− 0.00	0.75*
EAS emotionality	0.74*	0.17*	− 0.19*
EAS shyness	0.14*	**0**.81*	0.01
CCTI attention span	− 0.35*	− 0.03	0.55*
German sample^b^			
EAS emotionality	0.57*	0.09*	− 0.16*
EAS shyness	0.22*	**0**.78*	0.06
CCTI attention span	− 0.37*	0.01	0.67*
Chinese sample^c^			
CBQ anger	0.52*	0.09	− 0.17
CBQ shyness	0.10	0.65*	− 0.08
CBQ attentional focusing	− 0.30*	− 0.14	0.71*

Expected validity correlations are boldfaced

*ICTS* Integrative Child Temperament Screener, *CBQ-SF* Children’s Behavior Questionnaire-Short Form, *EAS* Emotionality, Activity, and Sociability Questionnaire, *CCTI* Colorado Child Temperament Inventory

*p < 0.001

^a^*N* (CBQ-SF) = 3153. *N* (EAS/CCTI) = 1067

^b^*N* = 1713

^c^*N* = 283. In China, the CBQ-SF was administered to an offline sample described in Study 2a. The findings are reported here for the sake of comparability with the validity correlations from other Study 1 samples

## Study 2: test–retest reliability and interrater agreement

Study 2a was conducted to examine the test–retest reliability and interrater agreement for the German, English, and Chinese versions of the ICTS. In addition, the convergent validity of the Chinese version was examined via the same CBQ-SF scales that were used for the same purpose in Study 1. To this end, three separate samples were recruited locally: in the German-speaking part of Switzerland, in the UK, and in China. The results are presented in Table 7, which shows the values for test–retest reliability and for parental agreement in all three samples. The retest correlations were satisfactory overall, and results for parent agreement were similar to findings from other studies (for samples and procedures, see Supplementary Materials 5).

As a corollary to the examination of parent agreement, interrater reliability was also explored across preschool teachers in a separate study (Study 2b). Three female daycare teachers were provided with the temperament questionnaire and asked to rate each of 20 children whom they saw on different days of the week. Teacher-to-teacher correlations averaged *r* = 0.55 and were thus in the same order of magnitude as the parent agreement (for samples, procedures, and more detailed results, see Supplementary Materials 6).

**Table 7:** Test–retest reliability and parental agreement

ICTS scales	Test–retest			Parental agreement		
	UK^a^	Germany^b^	China^c^	UK^d^	Germany^e^	China^f^
Frustration	0.77*	0.71*	0.74*	0.50*	0.46*	0.34*
Inhibition	0.79*	0.77*	0.77*	0.61*	0.65*	0.41*
Attention	0.81*	0.72*	0.72*	0.49*	0.54*	0.51*

*ICTS *Integrative Child Temperament Screener

*p < 0.001

^a^Average of mothers’ (*N* = 53) and fathers’ (*N* = 53) retest values

^b^Mothers provided retest (*N* = 144)

^c^Mothers provided retest (*N* = 53)

^d^*N* = 53 mothers and 53 fathers

^e^*N* = 191 mothers and 191 fathers

^f^*N* = 91 mothers and 91 fathers

## Study 3: associations with behavior problems and screening accuracy

Study 3 was conducted to examine the clinical validity of the ICTS by focusing on patterns of association between the ICTS dimensions and the Strengths and Difficulties Questionnaire (SDQ). A secondary goal was to evaluate critical bands and screening accuracy of the instrument.

### Methods

*Sample and procedure* The sample consisted of 404 children (251 boys, 153 girls) with a mean age of 4.91 years (*SD* 1.96). Caregiver ratings of the children’s temperament were obtained via a new website that was disseminated in the United Kingdom. In addition to the general information provided about child temperament on the welcome page, the introductory page also asked parents to rate their child for the presence of behavioral issues. The study was approved by the departmental ethics committee and participants provided informed consent before taking the survey.

### Measures

*Strengths and Difficulties Questionnaire (SDQ)* The SDQ is a 25-item questionnaire that provides scores for emotional symptoms, conduct problems, hyperactivity/inattention, peer problems, and prosocial behavior [62]. The four symptom scales are strongly related to the Achenbach Child Behavior Checklist and have been found to provide similar screening efficiency [63].

*Integrative Child Temperament Inventory* The full version of the instrument was administered (see Study 1, Methods), but analyses are confined to the items of the ICTS.

*Children’s Behavior Questionnaire-Short Form (CBQ-SF)* The CBQ-SF scales anger, shyness, and attentional focusing were administered to compare ICTS-to-SDQ with CBQ-SF-to-SDQ associations.

### Results

*Associations between ICTS dimensions and SDQ behavioral symptoms* The unique relationship between the ICTS scales and the four problem areas of the SDQ was examined by means of a multivariate regression. Consistent with predictions derived from the literature [34, 39, 47], ICTS frustration was the temperament scale most distinctively associated with SDQ conduct problems, ICTS attentional persistence was the scale most specifically associated with SDQ hyperactivity, and ICTS inhibition was the scale most distinctively associated with SDQ emotional symptoms (see Table 8). The SDQ also includes a prosocial behavior scale, and associations between the ICTS dimensions and prosocial behavior (also reported in Table 8) are consistent with previous research that found effortful control to be a strong predictor of mature and conscientious child behavior [64].

**Table 8:** Multiple regression. Unique contributions (standardized beta weights) of ICTS and CBQ-SF scales to SDQ behavioral problem and prosocial behavior scales, with child age and gender controlled for

SDQ symptom scales	ICTS temperament dimensions		
	Anger/Frustration	Attentional persistence	Behavioral inhibition
Conduct problems	0.54***	− 0.12**	− 0.05
Hyperactivity	0.20***	− 0.64***	− 0.06
Emotional symptoms	0.24***	− 0.01	0.43***
Peer problems	0.18***	− 0.09*	0.16**
Prosocial behavior	− 0.36***	0.11*	− 0.14**
Externalizing	0.43***	− 0.46***	0.03
Internalizing	0.22***	− 0.06	0.43***

	CBQ-SF temperament dimensions		
	Anger	Attentional focusing	Shyness
Externalizing	0.39***	− 0.48***	− 0.10**
Internalizing	0.23***	− 0.12**	0.39***

*ICTS* Integrative Child Temperament Screener, *SDQ* Strengths and Difficulties Questionnaire, *CBQ*-*SF* Children’s Behavior Questionnaire-Short Form, *N* = 404. Coefficients are standardized beta weights, representing unique contributions of each temperament dimension to problem scores, with child age and gender controlled for

*p < 0.05. **p < 0.01. ***p < 0.001

The ICTS-to-SDQ associations were similar to the CBQ-to-SDQ associations for both externalizing and internalizing symptoms, as can be seen from the lower part of Table 8. Although half as long as the CBQ-SF scales, the ICTS scales explained about the same amount of variance in children’s externalizing and internalizing behaviors (ICTS: 59% and 30%, respectively; CBQ-SF: 56% and 30%, respectively). To explore critical bands of the temperament scales and their receiver-operating characteristics, children were allocated to an externalizing or an internalizing behavior problem group in accordance with the SDQ scoring norms. These analyses, which are reported in Supplementary Materials 7, suggest that the ICTS offers a favorable balance between brevity and screening accuracy (AUCs .82 and .75 for externalizing and internalizing symptoms, respectively).

### Discussion

The current measure goes an important step beyond previously existing measures toward meeting the requirement for a tool that can identify early childhood temperament risk factors early, easily, and broadly. First, it is based on large samples collected in several different countries and on data from multiple informants (i.e., both parents and teachers). Second, to the author’s knowledge, it is the first measure of child temperament whose measurement invariance has been examined across many nations and age groups contemporaneously, thus putting the scale on a firm methodological and empirical footing. Third, by confining the measure’s coverage to well-researched child temperament traits with a consistent record of predicting behavior disorders up to adulthood, it was possible to create a tool that is very brief yet psychometric viable. Finally, the scale can be used for children as young as 2 years of age, when the relatively high degree of brain and behavioral plasticity gives interventions a better chance to succeed.

The current demonstration of invariance of ICTS factor loadings across age groups is particularly essential in light of the frequent need for comparing temperament-to-behavior problem associations at different time points in longitudinal research. Equivalence of factor loadings was also supported across countries, although conclusions are necessarily limited to the nations that were included in the current research. Equivalence of item intercepts was achieved in terms of partial, but not full, measurement invariance. Specifically, scalar invariance was demonstrated for two items per factor, giving researchers the option of using a reduced six-item scale for mean comparisons across ages or countries.

Although the advantages of brevity and practicality are obvious, brief scales often raise concerns about content validity. All while being rational, this concern does not bear deeper scrutiny in light of a number of current findings. First, gender differences obtained with the ICTS reproduced results that had previously been obtained with broader measures of temperament. Second, in Study 1, the pattern of convergent correlations with longer and more comprehensive temperament measures was in line with expectations in all of the countries and regardless of the type of validation measure used (EAS and CBQ-SF). Furthermore, associations with parental perceptions of child difficulty were highest for frustration and lowest for inhibition, with inattention falling in the middle, as has been found in other studies. Third, and perhaps most crucially, criterion-related associations were corroborated by a pattern of differential relationships between the three temperament scales of the ICTS and SDQ behavioral symptoms in Study 3, consistent with predictions derived from the literature. Study 3 also provided preliminary evidence concerning the instrument’s screening accuracy. It is noteworthy that the ICTS and the corresponding scales of the CBQ-SF used in this study explained considerably more variance in problem behaviors than has been reported for the higher order factors of the CBQ-SF and CBQ-VSF [66]. Finally, reliability indicators, such as test–retest reliability and interrater agreement, were in the range of psychometric properties reported for longer measures of child temperament (e.g., [55, 67].

## Implications and uses

The ICTS has potential applications in both research and applied contexts. In research settings, it allows investigators to collect basic information on temperament where this would have been difficult until now, notably in situations when time with participants is very limited, when numerous other constructs must be assessed, or when temperament needs to be included as a secondary or control variable. The advantages of brevity and practicality are supplemented by the measure’s suitability for cross-cultural and longitudinal research. In applied settings, the measure lends itself to a quick assessment of a child’s temperament in the context of screening for behavioral or emotional risk, such as in primary pediatric care, thus providing a diagnostic tool to match recent developments in temperament-focused interventions.

More specifically, the last decade has seen the advent of several temperament-based interventions that use parent and teacher guidance [68], behavioral skills training [69], and computer exercises aimed at promoting self-regulation (e.g., [70] or reducing behavioral inhibition (e.g., [71]. One advantage of using temperament concepts in screening contexts is that temperament refers to individual differences within the normal range. Thus, assessment and intervention can capitalize on a vocabulary that is relatively benign and accessible. Follow-ups to a positive screen may thus be more easily framed in terms of enhancing “character literacy” rather than preventing psychopathology or violence. These features could positively affect parents’, teachers’, and primary child care providers’ motivation to engage with apposite forms of counseling or intervention [72].

## Limitations

Results from the current research should be interpreted within its limitations. First, the ICTS was developed as an addition to and not as a replacement for longer, fine-grained measures of child temperament, of which there are already many excellent examples. Nor does the ICTS intend to include all child temperament dimensions that could potentially place a child at risk for behavior problems. As noted at the beginning, the selection of traits was guided by their predictive validity for behavior disorders over the long term and by the likelihood of their assessment exhibiting measurement invariance. As more birth cohort studies that include early temperament assessments come to maturity, additional traits may have to be included. Second, although the samples were comparatively large and diverse, they were biased toward children from educated backgrounds. Third, the ICTS was not administered separately from its parent instrument. This limitation is tempered by the similar performance of the nine items across several large independent national samples and age groups. Even so, conclusions about the ICTS as a stand-alone instrument should be considered preliminary. Fourth, the amount of validational information differed across the countries: Although it is reasonably extensive in the US, UK, Germany, and China, information relating to the Spanish language version is less complete, calling for additional studies to determine its merits in Spanish-speaking populations. Fifth, studies including clinical populations are needed to confirm the ICTS’s credentials as a screening tool. More generally, the validation of a psychological measure is a gradual, ongoing process for which the current studies provide a point of departure.

## Conclusion

The above limitations notwithstanding, the ICTS makes a unique addition to current temperament assessment tools by showing that temperament characteristics placing children at risk for developing behavior problems much later in life can be identified early, rapidly, and equivalently across countries and age groups. As such, the scale contributes to fill a gap in current screening tools for identifying behavioral and emotional risk factors in childhood.

### Electronic supplementary material

Below is the link to the electronic supplementary material.

Supplementary material 1 (DOCX 66 kb)

Supplementary material 2 (DOCX 16 kb)

Supplementary material 3 (DOCX 32 kb)

Supplementary material 4 (DOCX 15 kb)

Supplementary material 5 (DOCX 101 kb)

Supplementary material 6 (DOCX 79 kb)

Supplementary material 7 (DOCX 115 kb)

## Appendix

### Items of the integrative child temperament screener^1^

*Anger/Frustration Subscale (Frustration)*

(1) “Explodes if cannot have what he/she wants (e.g., a certain toy, candy, clothing)”

(2) “Cries or yells when asked to stop favorite occupation”

(3) “Is even-tempered, easy to manage” (R)

*Behavioral Inhibition Subscale (Inhibition)*

(1) “Approaches unfamiliar children and joins in their games” (R)

(2) “Hides behind mother (and/or another caretaker) when meeting unfamiliar people”

(3) “Is shy when meeting unfamiliar children”

*Attentional Persistence Subscale (Attention)*

(1) “Is easily distracted from his/her projects” (R)

(2) “When looking at a book or painting, is quickly bored and changes activity” (R)

(3) “Concentrates on something for long periods of time without difficulty (e.g., tasks, games, books)”

*Scoring instructions*

All items are presented on a 6-point scale ranging from *behavior occurs never or hardly ever* (1) to *behavior occurs always or close to always* (6). R indicates that the item needs reverse-scoring.
