# Supplementary material for: Identifying child temperament risk factors from 2 to 8 years of age: validation of a brief temperament screening tool in the US, Europe, and China
Source: Eur Child Adolesc Psychiatry. 2019 Aug 14;29(5):665–78. doi: 10.1007/s00787-019-01379-5 (PMC7250798; doi:10.1007/s00787-019-01379-5)
Supplement: Supplementary file 1 — Supplementary material 1 (DOCX 66 kb) [file 787_2019_1379_MOESM1_ESM.docx]

Supplementary Table 1

*Summary and Capsule Definitions of Three Clinically Significant Child Temperament Components and Related Dimensions*

| Temperament Component | Capsule definitions | Related dimensions | Authors/Model |
| --- | --- | --- | --- |
| Behavioral inhibition | Inhibition of behavior in response to novel unfamiliar people and situations | Harm avoidance  Shyness  Social fearfulness  Withdrawal | Cloninger [10]  Rothbart [11]; Buss & Plomin [9] Campos & Goldsmith [7, 69]  Thomas & Chess [6] |
| Irritability/frustration | Aggressive or irritated behavior in response to painful and/or frustrating input | Anger proneness  Anger  Difficultness Emotionality | Campos & Goldmsith [7, 67]  Rothbart [15]  Thomas & Chess [6] Buss & Plomin [9] |
| Attention/persistence | Capacity for attentional focusing and control as a basis for voluntary behavior, including persistence | Attentional control/focusing Effortful control Novelty seeking (-) Persistence | Rothbart (15)  Rothbart (15)  Cloninger [10]  Cloninger [10]; Chess & Thomas [6]  Campos & Goldsmith [7, 67] |

*Note.* Adapted from Zentner and Bates (2008).

^a^ A minus sign (-) indicates that the dimension is negatively related to the target dimension.
