# Supplementary material for: Identifying child temperament risk factors from 2 to 8 years of age: validation of a brief temperament screening tool in the US, Europe, and China
Source: Eur Child Adolesc Psychiatry. 2019 Aug 14;29(5):665–78. doi: 10.1007/s00787-019-01379-5 (PMC7250798; doi:10.1007/s00787-019-01379-5)
Supplement: Supplementary file 2 — Supplementary material 2 (DOCX 16 kb) [file 787_2019_1379_MOESM2_ESM.docx]

Supplementary Materials 2

*Level of Tertiary Education in the Current Samples and in Representative Populations of the Respective Countries*^a^

| Country | Current Samples | | Representative Populations^b^ |
| --- | --- | --- | --- |
|  | *N* | Percentage | Percentage |
| US | 3351 | 67.9 | 52.2 |
| UK | 417 | 57.2 | 53.6 |
| Germany | 1898 | 42.9 | 32.5 |
| Spain | 1115 | 63.2 | 48.8 |

^a^ Because of difficulties in obtaining comparable data for China, the latter is omitted.

^b^ Data for 25- to 34-year-old women in 2017. Source: OECD (2018), Population with tertiary education (indicator). doi:10.1787/0b8f90e9-en (Accessed on July 8, 2019).
