# Supplementary material for: Identifying child temperament risk factors from 2 to 8 years of age: validation of a brief temperament screening tool in the US, Europe, and China
Source: Eur Child Adolesc Psychiatry. 2019 Aug 14;29(5):665–78. doi: 10.1007/s00787-019-01379-5 (PMC7250798; doi:10.1007/s00787-019-01379-5)
Supplement: Supplementary file 3 — Supplementary material 3 (DOCX 32 kb) [file 787_2019_1379_MOESM3_ESM.docx]

**Supplementary Materials 3. Final Measurement Model: Parameter Estimates**

*Table 3a. Standardized Parameter Estimates for Partial Scalar Invariance for Age Groups*

| **Parameters** | Toddlers | Preschoolers | School-age children |
| --- | --- | --- | --- |
| **Factor Loadings** |  |  |  |
| **FRU** |  |  |  |
| FRU1 | 0.77 | 0.79 | 0.80 |
| FRU2 | 0.65 | 0.69 | 0.73 |
| FRU3 | 0.60 | 0.62 | 0.63 |
| **INH** |  |  |  |
| INH1 | 0.68 | 0.71 | 0.69 |
| INH2 | 0.52 | 0.55 | 0.54 |
| INH3 | 0.94 | 0.97 | 0.96 |
| **ATT** |  |  |  |
| ATT1 | 0.73 | 0.70 | 0.71 |
| ATT2 | 0.66 | 0.65 | 0.65 |
| ATT3 | 0.69 | 0.67 | 0.68 |
| **Covariances** |  |  |  |
| FRU ~INH | 0 | 0 | 0 |
| INH ~ATT | 0 | 0 | 0 |
| FRU ~ATT | -0.50 | -0.39 | -0.36 |
| **Intercepts** |  |  |  |
| FRU1 | 2.72 | 2.51 | 2.13 |
| FRU2 | 2.49 | 2.57 | 2.49 |
| FRU3 | 2.53 | 2.53 | 2.37 |
| INH1 | 2.04 | 2.01 | 2.01 |
| INH2 | 2.04 | 1.87 | 1.65 |
| INH3 | 1.85 | 1.80 | 1.83 |
| ATT1 | 2.65 | 2.55 | 2.42 |
| ATT2 | 2.71 | 2.68 | 2.53 |
| ATT3 | 2.45 | 2.59 | 2.65 |
| **Residuals** |  |  |  |
| FRU1 | 0.41 | 0.37 | 0.36 |
| FRU2 | 0.57 | 0.52 | 0.47 |
| FRU3 | 0.64 | 0.62 | 0.61 |
| INH1 | 0.54 | 0.49 | 0.53 |
| INH2 | 0.73 | 0.70 | 0.71 |
| INH3 | 0.11 | 0.06 | 0.09 |
| ATT1 | 0.47 | 0.51 | 0.50 |
| ATT2 | 0.57 | 0.58 | 0.57 |
| ATT3 | 0.54 | 0.55 | 0.53 |
|  |  |  |  |

*Table 3b. Unstandardized Parameter Estimates for Partial Scalar Invariance for Age Groups*

| **Parameters** | Toddlers | | Preschoolers | School-age children | | |
| --- | --- | --- | --- | --- | --- | --- |
| **Factor Loadings** |  |  | | |  | |
| **FRU** |  |  | | |  | |
| FRU1 | 1.00 | 1.00 | | | 1.00 |  |
| FRU2 | 0.87 | 0.87 | | | 0.87 |  |
| FRU3 | 0.77 | 0.77 | | | 0.77 |  |
| **INH** |  |  | | |  |  |
| INH1 | 1.00 | 1.00 | | | 1.00 |  |
| INH2 | 0.75 | 0.75 | | | 0.75 |  |
| INH3 | 1.38 | 1.38 | | | 1.38 |  |
| **ATT** |  |  | | |  |  |
| ATT1 | 1.00 | 1.00 | | | 1.00 |  |
| ATT2 | 0.95 | 0.95 | | | 0.95 |  |
| ATT3 | 1.05 | 1.05 | | | 1.05 |  |
| **Covariances** |  |  | | |  |  |
| FRU ~INH | 0.00 | 0.00 | | | 0.00 |  |
| INH ~ATT | 0.00 | 0.00 | | | 0.00 |  |
| FRU ~ATT | -0.50 | -0.44 | | | -0.48 |  |
| **Intercepts** |  |  | | |  |  |
| FRU1 | 4.02 | 3.73 | | | 3.39 |  |
| FRU2 | 3.80 | 3.80 | | | 3.80 |  |
| FRU3 | 3.70 | 3.70 | | | 3.70 |  |
| INH1 | 3.31 | 3.31 | | | 3.31 |  |
| INH2 | 3.25 | 2.99 | | | 2.58 |  |
| INH3 | 2.99 | 2.99 | | | 2.99 |  |
| ATT1 | 3.56 | 3.56 | | | 3.56 |  |
| ATT2 | 3.86 | 3.86 | | | 3.86 |  |
| ATT3 | 3.70 | 3.94 | | | 4.24 |  |
| **Residuals** |  |  | | |  |  |
| INH3 | 0.88 | 0.82 | | | 0.92 |  |
| FRU1 | 1.34 | 1.14 | | | 1.09 |  |
| FRU2 | 1.38 | 1.33 | | | 1.48 |  |
| FRU3 | 1.42 | 1.33 | | | 1.44 |  |
| INH1 | 1.83 | 1.77 | | | 1.73 |  |
| INH2 | 0.30 | 0.16 | | | 0.23 |  |
| ATT1 | 0.85 | 1.00 | | | 1.07 |  |
| ATT2 | 1.16 | 1.21 | | | 1.34 |  |
| ATT3 | 1.23 | 1.28 | | | 1.36 |  |
|  |  |  | | |  | |

*Table 3c. Standardized Parameter Estimates for Partial Scalar Invariance for Nation Groups*

| **Parameters** | US | Germany | China | Spain | UK | |
| --- | --- | --- | --- | --- | --- | --- |
| **Factor Loadings** |  |  |  |  |  |  |
| **FRU** |  |  |  |  |  |  |
| FRU1 | 0.67 | 0.79 | 0.71 | 0.78 | 0.84 |  |
| FRU2 | 0.58 | 0.66 | 0.66 | 0.71 | 0.79 |  |
| FRU3 | 0.51 | 0.59 | 0.61 | 0.57 | 0.63 |  |
| **INH** |  |  |  |  |  |  |
| INH1 | 0.63 | 0.70 | 0.69 | 0.60 | 0.67 |  |
| INH2 | 0.49 | 0.52 | 0.51 | 0.50 | 0.50 |  |
| INH3 | 0.99 | 0.99 | 0.99 | 1.00 | 1.00 |  |
| **ATT** |  |  |  |  |  |  |
| ATT1 | 0.70 | 0.70 | 0.70 | 0.63 | 0.70 |  |
| ATT2 | 0.67 | 0.69 | 0.70 | 0.61 | 0.70 |  |
| ATT3 | 0.71 | 0.71 | 0.65 | 0.70 | 0.70 |  |
| **Covariances** |  |  |  |  |  |  |
| FRU ~INH | 0.00 | 0.00 | 0.00 | 0.00 | 0.00 |  |
| INH ~ATT | 0.00 | 0.00 | 0.00 | 0.00 | 0.00 |  |
| FRU ~ATT | -0.42 | -0.35 | -0.44 | -0.35 | -0.41 |  |
| **Intercepts** |  |  |  |  |  |  |
| FRU1 | 2.82 | 3.03 | 2.67 | 2.61 | 2.54 |  |
| FRU2 | 2.76 | 2.86 | 2.81 | 2.66 | 2.71 |  |
| FRU3 | 2.64 | 2.81 | 3.29 | 2.34 | 2.21 |  |
| INH1 | 2.00 | 2.15 | 2.06 | 1.95 | 2.03 |  |
| INH2 | 1.86 | 1.92 | 1.81 | 1.95 | 1.81 |  |
| INH3 | 1.98 | 1.91 | 1.84 | 2.02 | 1.87 |  |
| ATT1 | 2.45 | 2.57 | 2.43 | 2.39 | 2.44 |  |
| ATT2 | 2.49 | 2.67 | 2.59 | 2.46 | 2.56 |  |
| ATT3 | 2.47 | 2.43 | 2.47 | 2.82 | 2.16 |  |
| **Residuals** |  |  |  |  |  |  |
| FRU1 | 0.55 | 0.37 | 0.50 | 0.39 | 0.30 |  |
| FRU2 | 0.66 | 0.56 | 0.57 | 0.50 | 0.37 |  |
| FRU3 | 0.74 | 0.65 | 0.62 | 0.67 | 0.61 |  |
| INH1 | 0.61 | 0.52 | 0.52 | 0.64 | 0.55 |  |
| INH2 | 0.76 | 0.73 | 0.74 | 0.75 | 0.75 |  |
| INH3 | 0.00 | 0.00 | 0.00 | 0.00 | 0.00 |  |
| ATT1 | 0.52 | 0.51 | 0.52 | 0.60 | 0.51 |  |
| ATT2 | 0.56 | 0.53 | 0.51 | 0.63 | 0.51 |  |
| ATT3 | 0.50 | 0.50 | 0.58 | 0.52 | 0.51 |  |

*Table 3d. Unstandardized Parameter Estimates for Partial Scalar Invariance for Nation Groups*

| **Parameters** | US | Germany | China | Spain | UK |
| --- | --- | --- | --- | --- | --- |
| **Factor Loadings** |  |  |  |  |  |
| **FRU** |  |  |  |  |  |
| FRU1 | 1.00 | 1.00 | 1.00 | 1.00 | 1.00 |
| FRU2 | 0.90 | 0.90 | 0.90 | 0.90 | 0.90 |
| FRU3 | 0.76 | 0.76 | 0.76 | 0.76 | 0.76 |
| **INH** |  |  |  |  |  |
| INH1 | 1.00 | 1.00 | 1.00 | 1.00 | 1.00 |
| INH2 | 0.75 | 0.75 | 0.75 | 0.75 | 0.75 |
| INH3 | 1.49 | 1.49 | 1.49 | 1.49 | 1.49 |
| **ATT** |  |  |  |  |  |
| ATT1 | 1.00 | 1.00 | 1.00 | 1.00 | 1.00 |
| ATT2 | 1.03 | 1.03 | 1.03 | 1.03 | 1.03 |
| ATT3 | 1.10 | 1.10 | 1.10 | 1.10 | 1.10 |
| **Covariances** |  |  |  |  |  |
| FRU ~INH | 0.00 | 0.00 | 0.00 | 0.00 | 0.00 |
| INH ~ATT | 0.00 | 0.00 | 0.00 | 0.00 | 0.00 |
| FRU ~ATT | -0.40 | -0.34 | -0.46 | -0.39 | -0.54 |
| **Intercepts** |  |  |  |  |  |
| FRU1 | 4.06 | 4.06 | 4.06 | 4.06 | 4.06 |
| FRU2 | 4.12 | 4.12 | 4.12 | 4.12 | 4.12 |
| FRU3 | 3.81 | 3.82 | 4.36 | 3.75 | 3.59 |
| INH1 | 3.38 | 3.38 | 3.38 | 3.38 | 3.38 |
| INH2 | 3.00 | 3.00 | 3.00 | 3.00 | 3.00 |
| INH3 | 3.11 | 3.11 | 3.11 | 3.11 | 3.11 |
| ATT1 | 3.43 | 3.43 | 3.43 | 3.43 | 3.43 |
| ATT2 | 3.74 | 3.74 | 3.74 | 3.74 | 3.74 |
| ATT3 | 3.73 | 3.51 | 4.10 | 4.04 | 3.37 |
| **Residuals** |  |  |  |  |  |
| INH3 | 0.00 | 0.00 | 0.00 | 0.00 | 0.00 |
| FRU1 | 1.14 | 0.67 | 1.16 | 0.94 | 0.76 |
| FRU2 | 1.47 | 1.16 | 1.22 | 1.20 | 0.86 |
| FRU3 | 1.54 | 1.21 | 1.09 | 1.72 | 1.60 |
| INH1 | 1.74 | 1.27 | 1.40 | 1.94 | 1.52 |
| INH2 | 2.00 | 1.78 | 2.03 | 1.77 | 2.07 |
| ATT1 | 1.01 | 0.91 | 1.02 | 1.24 | 1.00 |
| ATT2 | 1.26 | 1.04 | 1.06 | 1.45 | 1.09 |
| ATT3 | 1.12 | 1.04 | 1.58 | 1.06 | 1.25 |

Note: FRU= Anger/Frustration; INH = Behavioral Inhibition; ATT = Attentional Persistence
