# Supplementary material for: Identifying child temperament risk factors from 2 to 8 years of age: validation of a brief temperament screening tool in the US, Europe, and China
Source: Eur Child Adolesc Psychiatry. 2019 Aug 14;29(5):665–78. doi: 10.1007/s00787-019-01379-5 (PMC7250798; doi:10.1007/s00787-019-01379-5)
Supplement: Supplementary file 4 — Supplementary material 4 (DOCX 15 kb) [file 787_2019_1379_MOESM4_ESM.docx]

Supplementary Materials 4

*Parental Difficulty Perceptions: Scale Means, Standard Deviations, and Correlations with ICTS Temperament Dimensions*

| Country | Mean (*SD*) | Correlations With Difficulty Score | | |
| --- | --- | --- | --- | --- |
|  |  | Frustration | Inhibition | Attention |
| US | 3.63 (.98) | .65* | .03 | -.35* |
| UK | 3.54 (1.04) | .66* | .03 | -.36* |
| Germany | 3.20 (.96) | .54* | .02 | -.40* |
| Spain | 3.15 (.97) | .69* | -.01 | -.38* |
| China | 2.96 (.74) | .41* | .15* | -.31* |

*Note.* ICTS = Integrative Child Temperament Screener.

**p* < .001
