# Supplementary material for: Identifying child temperament risk factors from 2 to 8 years of age: validation of a brief temperament screening tool in the US, Europe, and China
Source: Eur Child Adolesc Psychiatry. 2019 Aug 14;29(5):665–78. doi: 10.1007/s00787-019-01379-5 (PMC7250798; doi:10.1007/s00787-019-01379-5)
Supplement: Supplementary file 5 — Supplementary material 5 (DOCX 101 kb) [file 787_2019_1379_MOESM5_ESM.docx]

**Supplementary Materials 5**

**Study 2**

**Sample and Procedures**

**German-speaking sample.** Participants were parents of 191 children (96 boys, 95 girls), who were rated by both parents at Time 1. The average age of the boys was 48.23 months (*SD* 20.03) and of the girls was 56.23 months (*SD* 24.70). They were invited to participate in the study via ads in a local newspaper and were mailed a copy of the questionnaire and instructions on how to complete the ratings. Parents were asked to fill in and return the questionnaire independently in a self-stamped envelope within 2 weeks and to indicate whether they were able to fill in the questionnaire a second time. Participants who returned the questionnaire were given a link to a website and asked to fill in the questionnaire a second time online. Because the vast majority of participants consenting to provide a second rating were mothers, only mothers were invited to participate in the retest part. A total of 184 children (94 girls, 90 boys) were rated again after an average interval of 14 days.

**English-speaking sample.** Parents were recruited through mailings in Northern England, using the same procedure as with the German-speaking sample. At Time 1, the sample consisted of 53 children (25 boys, 28 girls), who were rated by both parents. The average age of the boys was 57.12 months (*SD* 29.88) and of the girls was 70.68 months (*SD* 33.48). Seventeen children (11 boys, 6 girls) were rated again after an average interval of 14 days by both parents.

**Chinese sample.** Parents of 283 children (146 boys, 137 girls) were recruited through two kindergartens in Shenzhen, China. Parents were informed about the purpose of the study through the kindergarten teachers. Interested parents collected envelopes from the kindergarten teachers that included copies of the questionnaires, informed consent forms, and instructions on how to complete the ratings. Parents were asked to return the questionnaires to the kindergarten teacher in a sealed envelope within 1 week and to state whether they would be interested in participating in a retest session.

Ratings from both parents were obtained for 91 of the children (48 boys, 43 girls), whereas all the other children were rated by one parent only. The average age of the boys was 52.97 months (*SD* 9.39) and of the girls was 53.09 months (*SD* 9.16). Thirty-nine mothers and 14 fathers agreed to participate in the retest session and provided retest ratings of 53 children (31 boys, 22 girls). The average interval between the first and the second rating was 22 days.
