# Supplementary material for: Identifying child temperament risk factors from 2 to 8 years of age: validation of a brief temperament screening tool in the US, Europe, and China
Source: Eur Child Adolesc Psychiatry. 2019 Aug 14;29(5):665–78. doi: 10.1007/s00787-019-01379-5 (PMC7250798; doi:10.1007/s00787-019-01379-5)
Supplement: Supplementary file 6 — Supplementary material 6 (DOCX 79 kb) [file 787_2019_1379_MOESM6_ESM.docx]

**Supplementary Materials 6**

**Study 3**

**Sample and Procedures**

Participants were 20 children aged 2 to 5 years and three female caregivers (Caregivers 1, 2, and 3) from a university campus nursery, aged 37, 49, and 38 years, respectively. Caregiver 1 had 11 years of childcare experience (7 of which had been spent in the nursery), Caregiver 2 had 25 years of childcare experience (19 years at the nursery), and Caregiver 3 had 20 years of experience (3.5 years at the nursery). All caregivers had known each child from the age of 2, when the children entered the nursery. They had weekly contact with each of the 20 children that they agreed to rate, and each child was rated by all three caregivers, resulting in a total of 60 returned rating forms. The questionnaire handed to the teachers consisted of a shortened version of the original ICTI scales that included four items per dimension. For the purpose of comparison, the analyses below are confined to the 3 ICTS items per dimension.

**Results**

*Teacher by Teacher Interrater Agreement*

| ICTS Scales | Composite Reliability |  | Teacher-to-Teacher Correlations | | |
| --- | --- | --- | --- | --- | --- |
|  | All Teachers  (ICC) |  | Teacher 1  Teacher 2 | Teacher 1  Teacher 3 | Teacher 2  Teacher 3 |
| Frustration | .83 |  | .61 | .71 | .59 |
| Inhibition | .77 |  | .52 | .73 | .41 |
| Attention | .71 |  | .76 | .35 | .26 |

*Note*: ICTS = Integrative Child Temperament Screener; ICC = intraclass correlation coefficient.
