# Supplementary material for: Identifying child temperament risk factors from 2 to 8 years of age: validation of a brief temperament screening tool in the US, Europe, and China
Source: Eur Child Adolesc Psychiatry. 2019 Aug 14;29(5):665–78. doi: 10.1007/s00787-019-01379-5 (PMC7250798; doi:10.1007/s00787-019-01379-5)
Supplement: Supplementary file 7 — Supplementary material 7 (DOCX 115 kb) [file 787_2019_1379_MOESM7_ESM.docx]

**Supplementary Material 7**

**Clinical Relevance of the ICTS.** In the subsequent analyses, the SDQ symptom scales were combined into externalizing and internalizing problem scales, following the recommendations by Goodman, Lamping, and Ploubidis [65]. Critical bands on the temperament scales were explored by forming two subclinical groups, one for externalizing and one for internalizing problems, in accordance with the SDQ scoring norms published on the SDQ website ([www.sdqinfo.org/py/sdqinfo/c0.py)](http://www.sdqinfo.org/py/sdqinfo/c0.py)). Thus, children whose scores fell into the high (previously called abnormal) range of the distribution of the respective SDQ dimensions were allocated to these two groups, resulting in an externalizing risk group (*N* = 71) and an internalizing risk group (*N* = 93).

Subsequently, the distributions of the temperament scales for the symptomatic and non-symptomatic children were compared. Because internalizing symptoms are distinctly predicted by inhibition, whereas externalizing symptoms are predicted by both frustration and inattention, behavioral inhibition was treated as the sole temperament risk factor for internalizing problems, whereas frustration and attentional focusing (reverse-scored) were aggregated into a second temperament risk factor for externalizing disorder. This factor was termed “impulsivity” in light of both constituent variables’ relationship with low self-control. The receiver operating characteristic curves showed good screening accuracy of the impulsivity composite for externalizing problem behaviors (AUC = .82) and fair screening accuracy for the inhibition subscale (AUC = .75). Additional details of the sensitivity analyses are reported in Supplementary Tables 4a and 4b.

Supplementary Table 4a

*t-Test Comparisons and Area Under the Curves (AUCs) of ICTS-Impulsivity^a^ and of ICTS-Inhibition for Children at Risk and at No Risk for Externalizing and Internalizing Problems*

|  | Externalizing  Mean (*SD*) | No Externalizing  Mean (*SD*) | *t-*Test | *p-*Value | AUC (95% CI) |
| --- | --- | --- | --- | --- | --- |
| ICTS- |  |  |  |  |  |
| Impulsivity^a^ | 27.93 (4.13) | 21.24 (5.87) | 9.10 | <.001 | .82 (.77-.87) |
|  |  |  |  |  |  |
|  | Internalizing  Mean (*SD*) | No Internalizing  Mean (*SD*) |  |  |  |
| ICTS- |  |  |  |  |  |
| Inhibition | 12.46 (3.84) | 8.80 (3.79) | 8.12 | <.001 | .75 (.70-.81) |

*Note.* ICTS = Temperament Screener for Children; CI = confidence interval.

^a^The attentional focusing scale was reverse-scored and aggregated with the frustration scale to form a single risk temperament scale for externalizing problems called impulsivity (see text).

Supplementary Table 4b

*Positive and Negative Diagnostic Likelihood Ratios (LRs) for the Two Temperament Scale Values at Two Points of the Distribution*

|  | Externalizing Group | | Internalizing Group | | | |
| --- | --- | --- | --- | --- | --- | --- |
|  | ICTS Impulsivity^a^ | | ICTS Inhibition | | | |
|  | 50^th^ percentile | 66^th^ percentile | 50^th^ percentile | | 66^th^ percentile | |
|  |  |  |  | | |  |
| Score | 28.00 | 29.00 | 13.00 | 15.00 | | |
| LR+ | 4.34 | 4.94 | 3.36 | 4.14 | | |
| LR - | 0.55 | 0.67 | 0.61 | 0.76 | | |

*Note*. ICTS = Temperament Screener for Children; LR+ = positive likelihood ratio; LR- = negative likelihood ratio. ^a^See Supplementary Table 4a.
